# Pembrolizumab and epigenetic modification with azacitidine reshapes the tumor microenvironment of platinum-resistant epithelial ovarian cancer: a phase 2 non-randomized clinical trial

**DOI:** 10.1038/s43856-026-01404-0

**Published:** 2026-02-06

**Authors:** Blair V. Landon, Julia L. Boland, Andrea E. Wahner Hendrickson, Deborah K. Armstrong, Boris Winterhoff, Jaime Wehr, Akshaya V. Annapragada, Christopher Cherry, Archana Balan, Guneet Kaleka, Victor E. Velculescu, Stephen B. Baylin, Cynthia A. Zahnow, Dennis J. Slamon, Gottfried E. Konecny, Valsamo Anagnostou, John A. Glaspy

**Affiliations:** 1 Department of Oncology, Sidney Kimmel Comprehensive Cancer Center, Johns Hopkins University School of Medicine, Baltimore, MD USA; 2 Department of Medicine, Division of Internal Medicine, David Geffen School of Medicine, University of California Los Angeles, Los Angeles, CA USA; 3 Department of Medical Oncology, Mayo Clinic, Rochester, MN USA; 4 Department of Obstetrics and Gynecology, University of Minnesota, Minneapolis, MN USA; 5 Department of Medicine, UCLA-Olive View Medical Center, Sylmar, CA USA; 6 Department of Epigenetics, Van Andel Institute, Grand Rapids, MI USA; 7 Department of Medicine, Division of Hematology/Oncology, David Geffen School of Medicine, University of California Los Angeles, Los Angeles, CA USA

**Keywords:** Ovarian cancer, Tumour biomarkers, Tumour immunology, Epigenetics

## Abstract

**Background:**

Epigenetic modulators may sensitize platinum-resistant ovarian cancer (PROC) to immune checkpoint inhibition by reprogramming the tumor microenvironment.

**Methods:**

We report clinical and translational findings from a phase II non-randomized study of pembrolizumab and oral azacitidine in 34 women with PROC (NCT02900560). Key eligibility criteria included age 18 years or older, performance status of 0–1, measurable disease, platinum-resistant disease and histologically confirmed epithelial ovarian cancer, fallopian tube carcinoma or primary peritoneal carcinoma. Primary endpoints included safety, tolerability, overall response rate (ORR) and disease control rate (DCR). Secondary endpoints included CA-125 response. The effect of combined epigenetic and immunotherapy was evaluated by transcriptomic analyses of 72 serially biopsied tumors.

**Results:**

We show that the combination is moderately well tolerated and most common grade 3–4 adverse events are gastrointestinal side effects and anemia. ORR is 2.9% and DCR is 50%; with 3 of the 27 evaluable patients attaining a CA-125 response. Differential gene expression analyses reveal an upregulation of inflammatory and cytolytic genes and co-inhibitory checkpoints 6 weeks on-therapy. Upregulation of interferon signaling, antigen presentation and immune cell adhesion and migration gene sets are prominent on-therapy, together with an increase in density of CD8 + T-cells. Patients with a CA-125 and/or clinical response show an enrichment of adaptive and conserved immune response gene sets on-therapy.

**Conclusions:**

Our findings highlight the potential of epigenetic modulators to re-shape the tumor microenvironment of PROC toward a more inflammed phenotype and may point to approaches to augment immunotherapy response.

## Abstract (web summary)

Landon, Boland et al. investigate the clinical and molecular impact of a combination of epigenetic modulation and immune checkpoint inhibition in platinum-resistant ovarian cancer. Serial transcriptomic analyses reveal reshaping of the tumor microenvironment toward a more inflammatory state that may enhance immunotherapy response.

## Plain language summary

A type of cancer immunotherapy called immune checkpoint inhibition (ICI) has shown limited effectiveness in ovarian cancer. This might be because of few active immune cells in the area surrounding the tumor, called the tumor microenvironment. Additional treatments called epigenetic modulators can be used to increase anti-tumor immune responses. Here we investigate if the combination of an epigenetic modulator with ICI can be used in women with ovarian cancer who have not significantly benefited from standard chemotherapy. The combination of epigenetic therapy with ICI was moderately well tolerated, and tumor growth was controlled in half of the women in the study. There was evidence that the tumor microenvironment changed by inducing an inflammatory profile. These findings show the potential of this combination therapy to alter the tumor microenvironment, and that this could enhance outcomes to cancer treatments.

## Introduction

Ovarian cancer is the fifth leading cause of cancer-related death among women in the United States, and the leading cause of gynecologic cancer death in developed countries^1^. Most patients are diagnosed with advanced disease, and treatment options are limited for those with platinum-resistant epithelial ovarian cancer (PROC). Single agent anti-programmed cell death protein 1 (PD-1) or anti-programmed cell death ligand 1 (PD-L1) checkpoint inhibitor immunotherapy have limited effectiveness, with objective response rates of 8–10%^2,3^. This is in part attributed to ovarian cancer being considered a “cold tumor,” with decreased tumor-infiltrating lymphocytes (TILs) compared to other tumor types^4,5^. Additionally, tumor mutation burden (TMB), the prototypic measure of tumor foreignness and immune recognition is relatively low in ovarian cancer, with a mean TMB of 5.3 mutations/Mb in microsatellite stable tumors^6^.

Due to the cold tumor microenvironment of ovarian cancer, there has been an increased interest in utilizing epigenetic modulation to potentially sensitize ovarian cancer to immune checkpoint inhibition (ICI). DNA hypomethylating agents (HMAs) have shown promise in reshaping the TME of cold tumors into a more inflamed phenotype, which may be exploited to prime or re-sensitize tumors to immune checkpoint inhibitors^7–12^. One such epigenetic modulator, azacitidine (5-AZA) is a pyrimidine nucleoside analog of cytidine, which inhibits DNA methyltransferases (DNMTi’s), thereby blocking methylation in newly replicated DNA^13^. In addition to de-methylation of DNA hypermethylated promoters leading to re-expression of silenced tumor suppressor genes^13^, 5-AZA can reactivate transcription from repetitive elements of DNA and particularly human endogenous retrovirus (ERV) sequences^8–12,14,15^. The latter has been shown to lead to nucleic acid molecule sensing, activation of an IFN-inducing cellular response and trigger an innate antiviral response (a phenomenon termed “viral mimicry”)^9,10^. These findings supported an immunomodulatory role of epigenetic therapy with a potential to enhance the efficacy of cancer immunotherapy through the induction of innate immune responses. Consistent with this notion, in a pre-clinical model of melanoma, HMAs sensitized tumors to anti-cytotoxic T-lymphocyte associated protein 4 (CTLA-4) therapy^9^.

Similarly, in the clinical setting, 5-AZA has been shown to attract CD8 T-cells into the tumor bed of non-small cell lung cancer^16^. Notably, following disease progression on anti-PD-1 treatment, combining anti-PD-1 inhibition with 5-AZA reversed ICI resistance in some patients with non-small cell lung cancer supporting the notion that epigenetic therapy may re-sensitize ICI-resistant tumors^17^. In patients with chemotherapy-resistant ovarian cancer, use of HMAs may reverse platinum resistance^18^ and epigenetic priming with guadecitabine may induce anti-tumor immune responses, reflected in clinical benefit with sequential epigenetic therapy and ICI for patients with PROC^14^. Similar findings have been reported for patients with myelodysplastic syndromes, where the immune checkpoints PD-1 and CTLA-4 have been found to be upregulated with HMA therapy^19^ and combination of the DNMTi, guadecitabine, with the anti-PD-L1 agent, atezolizumab has been shown to drive clinical benefit^20^.

Taken together, preclinical and clinical evidence across cancer lineages suggests that combining ICI with hypomethylating agents may sensitize immunologically cold tumors, such as PROC, to ICI and improve the efficacy and objective responses compared to ICI alone. Here, we explore this notion in an open-label, phase II clinical trial of combined oral 5-AZA with pembrolizumab for patients with PROC, together with an in-depth evaluation of the effect of epigenetic modulation through serial comprehensive transcriptomic analyses of baseline and on-therapy tumors. This combined therapy regimen is moderately well tolerated and transcriptomic findings provide evidence of TME reshaping after the initiation of combination therapy. This combination therapy could represent a potential strategy for sensitizing PROC to ICI.

## Methods

### Study design, eligibility and participants

In this phase II study, eligible patients had a histologically confirmed diagnosis of epithelial ovarian cancer (EOC), fallopian tube carcinoma or primary peritoneal carcinoma. Other inclusion criteria included age greater than 18 years old, Eastern Cooperative Oncology Group (ECOG) performance status of less than or equal to 1, measurable disease per Immune-Related Response Evaluation Criteria in Solid Tumors (irRECIST), platinum-resistant or platinum-refractory disease, and patients must have received debulking surgery and pre- or post-operative platinum-based frontline chemotherapy. Patients with a platinum-free interval of less than 6 months from completion of a minimum of 4 platinum frontline therapy cycles were considered to have platinum-resistant disease, and patients who progressed while receiving frontline platinum-based chemotherapy were considered to have platinum-primary refractory disease. Patients must have had a tumor lesion amenable to biopsy and be willing to undergo two biopsies (baseline and at 6 weeks after the first treatment). The anatomic location of the two biopsies was not required to be the same lesion. The primary endpoint included safety and tolerability, along with immune-related overall response rate (ORR) and immune-related disease control rate (DCR) per irRECIST. The secondary endpoint was to assess the CA-125 response. CA-125 response was defined as a reduction of CA-125 of ≥ 50% from baseline assessment and clinical response was defined as a best overall response of stable disease or better based on irRECIST assessment and a treatment duration of ≥ 6 months. The original study contained a Part B, with its primary objective to compare the response and safety of the four cohorts, however it was terminated due to lack of efficacy. Patient characteristics and outcomes are summarized in Table 1. The study protocol and all amendments were approved by the institutional review boards (IRBs) of the participating institutions: UCLA IRB, University of Minnesota IRB, Mayo Clinic IRB, and Johns Hopkins IRB. Written informed consent was obtained by all patients in the study. Toxicity was assessed utilizing the National Cancer Institute Common Terminology Criteria for Adverse Events (NCI CTCAE) v4.03, and percentage of adverse events are recorded in Table 2. The study was registered in ClinicalTrials.gov, registration ID NCT02900560. The full eligibility criteria are listed below.

**Table 1:** Cohort demographic, clinicopathologic characteristics, radiographic response and clinical outcomes

Overall (*N* = 34)	(Number, %)
Age	
Mean (Standard deviation)	62 (10.9)
Median (Min and max)	63 (32–78)
Baseline ECOG performance status	
0	20 (59)
1	14 (41)
Histopathological diagnosis	
High-grade serous carcinoma	28 (82.4)
Clear cell carcinoma	4 (11.8)
Mixed	1 (2.9)
Endometrioid	1 (2.9)
FIGO stage at diagnosis	
IIIB	3 (8.8)
IIIC	16 (47.1)
IVA	3 (8.8)
IVB	11 (32.4)
Missing	1 (2.9)
Disease Status	
Platinum-Resistant	27 (79.4)
Platinum-Refractory	7 (20.6)
Number of Prior Lines in Platinum-Resistant/Refractory Setting	
2	19 (55.9)
3 or more	10 (29.4)
Missing	5 (14.7)
Best Overall Response	
Complete response	0 (0)
Partial response	1 (2.9)
Stable disease	16 (47.1)
Progressive disease	12 (35.3)
Not evaluable	5 (14.7)
ORR (%)	2.9
DCR (%)	50

*ORR* overall response rate, *DCR* disease control rate, *ECOG* Eastern Cooperative Oncology Group, *FIGO* Federation Internationale de Gynecologie et d’Obstetrique.

**Table 2:** Treatment-related adverse events in the study population

Adverse Effect	All (*n* = 32)		Cohort 1 (*n* = 7)		Cohort 2 (*n* = 10)		Cohort 3 (*n* = 7)		Cohort 4 (*n* = 8)	
	Any grade, No. (%)	Grade 3/4, No. (%)	Any grade, No. (%)	Grade 3/4, No. (%)	Any grade, No. (%)	Grade 3/4, No. (%)	Any grade, No. (%)	Grade 3/4, No. (%)	Any grade, No. (%)	Grade 3/4, No. (%)
Fatigue	12 (37.5)	4 (12.5)	5 (71.4)	1 (14.3)	3 (30)	1 (10)	6 (85.7)	1 (14.3)	7 (87.5)	1 (12.5)
Diarrhea	18 (56.3)	1 (3.1)	3 (42.9)	0	4 (40)	0	4 (57.1)	1 (14.3)	7 (87.5)	0
Vomiting	26 (81.3)	2 (6.3)	5 (71.4)	0	6 (60)	0	7 (100)	1 (14.3)	8 (100)	1 (12.5)
Nausea	24 (75)	1 (3.1)	5 (71.4)	0	6 (60)	0	5 (71.4)	0	7 (87.5)	1 (12.5)
Myalgia	4 (12.5)	0	1 (14.3)	0	1 (10)	0	1 (14.3)	0	1 (12.5)	0
Neutropenia	9 (28.1)	8 (25)	0	0	2 (20)	2 (20)	4 (57.1)	3 (42.9)	3 (37.5)	3 (37.5)
Thrombocytopenia	4 (12.5)	0	1 (14.3)	0	2 (20)	0	1 (14.3)	0	0	0
Anemia	13 (40.6)	4 (12.5)	3 (42.9)	2 (28.6)	3 (30)	2 (20)	3 (42.9)	0	4 (50.0)	0
Dehydration	5 (15.6)	0	2 (28.6)	0	0	0	1 (14.3)	0	2 (25.0)	0
AST	8 (25.0)	1 (3.1)	4 (57.1)	0	1 (10)	0	0	0	2 (25.0)	1 (12.5)
ALT	6 (18.8)	2 (6.3)	3 (42.9)	1 (14.3)	1 (10)	0	0	0	2 (25.0)	1 (12.5)
Abdominal Pain	7 (21.9)	0	2 (28.6)	0	3 (30)	0	1 (14.3)	0	1 (12.5)	0
Headache	6 (18.8)	1 (3.1)	2 (28.6)	0	0	0	3 (42.9)	0	1 (12.5)	1 (12.5)

*AST* aspartate aminotransferase elevation, *ALT* alanine aminotransferase elevation.

Inclusion Criteria

1. Signed and dated informed consent document obtained prior to initiation of any study specific procedure and treatment (by the subject or a legally acceptable representative as per the local regulations).

2. Women ≥ 18 years old.

3. Histologically confirmed epithelial ovarian cancer, fallopian tube carcinoma or primary peritoneal carcinoma.

4. Subjects must have received debulking surgery and preoperative and/or postoperative platinum-based frontline chemotherapy (intravenous and/or intraperitoneal) for the treatment of epithelial ovarian cancer, fallopian tube carcinoma or primary peritoneal carcinoma.

5. Subjects must have documented platinum-resistant or platinum-refractory disease. Platinum-resistant disease is defined as progression within <6 months from completion of a minimum of 4 platinum frontline therapy cycles in the pre or postoperative setting (the date should be calculated from the last administered dose of platinum agent). Platinum-refractory is defined as disease that has recurred/progressed while receiving platinum-based frontline therapy.

6. Measurable disease according to irRECIST for Part A. For Part B, subjects must have either measurable and/or non-measurable disease according to irRECIST.

7. Indication of systemic treatment for the relapsed epithelial ovarian cancer, fallopian tube carcinoma or primary peritoneal carcinoma.

8. For Part A, subjects must have a tumor lesion that is amenable to an image-guided core biopsy and willingness to undergo two biopsies (baseline and 6 weeks after first dose of study treatment). For Part B, subjects will be eligible even if their disease is not amenable to biopsy and/or the subject does not consent to the optional biopsies.

9. Eastern Cooperative Oncology Group (ECOG) Performance status 0 or 1.

10. Expected survival of more than 6 months.

11. Adequate organ function within 14 days prior to registration in Part A or randomization in Part B, as defined by the following criteria:

- Absolute neutrophils count (ANC) ≥ 1.5 ×10^9^/L, platelets ≥ 100 ×10^9^/L, hemoglobin > 9 g/dL (without transfusion or erythropoiesis stimulating agents dependency).
- Serum creatinine ≤ 1.5 x upper limit of normal (ULN).
- Total serum bilirubin ≤ 1.5 x ULN regardless of liver involvement secondary to tumor. Higher levels are acceptable if these can be attributed to active hemolysis or ineffective erythropoiesis.
- Serum aspartate transaminase (AST) and serum alanine transaminase (ALT) < 2.0 x ULN or ≤ 5 X ULN for subjects with liver metastases.
- International Normalized Ratio (INR) or Prothrombin Time (PT) ≤ 1.5 X ULN unless subject is receiving anticoagulant therapy as long as PT or PTT is within therapeutic range of intended use of anticoagulants.
- Partial Thromboplastin Time(PTT) or Activated Partial Thromboplastin Time (aPTT) ≤ 1.5 X ULN unless subject is receiving anticoagulant therapy as long as PT or PTT is within therapeutic range of intended use of anticoagulants.

12. For women of childbearing potential, negative serum pregnancy test within 14 days of registration in Part A or randomization in Part B.

13. Women of childbearing potential must agree to use acceptable methods of birth control throughout the study and up to 3 months after the last dose of study treatment. Recommendation is for 2 effective contraceptive methods during the study. Adequate forms of contraception are double-barrier methods (condoms with spermicidal jelly or foam and diaphragm with spermicidal jelly or foam), oral Depo Provera, or injectable contraceptives, intrauterine devices, and tubal ligation.

14. Willingness and ability to comply with scheduled visits, treatment plan, laboratory tests, and other trial procedures.

Exclusion Criteria

1. Non-epithelial ovarian cancers, including malignant mixed Müllerian tumors.

2. Ovarian tumors with low malignant potential (i.e. borderline tumors).

3. Subjects with relapse/progression based solely on elevation of CA-125, in absence of measurable disease (for Part A) or in the absence of measurable/non-measurable disease (for Part B), according to irRECIST criteria.

4. More than 2 prior treatment regimens for the platinum-resistant/refractory relapsed epithelial ovarian cancer, fallopian tube carcinoma or primary peritoneal carcinoma, defined as investigational, chemotherapy, hormonal, biologic, or targeted therapy.

5. Any concurrent or previous malignancy within 5 years prior to randomization (or registration in Part A) except for adequately and radically treated basal or squamous skin cancer, or carcinoma in situ of the cervix, or other non-invasive/in-situ neoplasm. A subject with previous history of invasive malignancy (other than adequately and radically treated basal or squamous skin cancer or carcinomas in situ) is eligible provided that she has been disease free for more than 5 years.

6. Subjects who have had radiotherapy or systemic anticancer therapy (investigational, chemotherapy, hormonal, biologic, or targeted) within 4 weeks prior to registration in Part A or randomization in Part B; or any ongoing acute clinically significant (as per investigator judgment) toxic effect of prior local or systemic anticancer therapy or any persisting complication of prior surgery.

7. Brain metastases (even if treated and/or stable), spinal cord compression, carcinomatous meningitis, or leptomeningeal disease.

8. Current or prior history of myelodysplastic syndrome, leukemia or clinically significant (as per investigator judgment) bone marrow failure.

9. Treatment with chronic systemic corticosteroid therapy ( > 10 mg/day of prednisone or equivalent) or any other form of immunosuppressive therapy within 2 weeks prior to registration in Part A or randomization in Part B.

10. Subject has an active autoimmune disease or a history of autoimmune disease or syndrome that has required systemic treatment in the past 2 years (i.e. with use of disease modifying agents, corticosteroids or immunosuppressive drugs). Replacement therapy (eg., thyroxine, insulin, or physiologic corticosteroid replacement therapy for adrenal or pituitary insufficiency, etc.) is not considered a form of systemic treatment. Subjects with vitiligo or resolved childhood asthma/atopy will not be excluded.

11. Subjects that received live vaccines within 30 days prior to registration in Part A or randomization in Part B.

12. Serious active infection requiring intravenous treatment and/or hospitalization at time of randomization (or of registration in Part A).

13. Has a known history of active TB (Bacillus Tuberculosis).

14. Known HIV infection or known positivity for active Hepatitis B (HBsAg reactive) or Hepatitis C (HCV RNA [qualitative] is detected).

15. Has known history of, or any evidence of active, non-infectious pneumonitis.

16. Has a history or current evidence of any condition, therapy, or laboratory abnormality that might confound the results of the trial, interfere with the subject’s participation for the full duration of the trial, or is not in the best interest of the subject to participate, in the opinion of the treating investigator.

17. Any contraindication to oral agents or significant nausea and vomiting, malabsorption, or significant small bowel resection that, in the opinion of the investigator, would preclude adequate absorption.

18. Known or suspected active drug or alcohol abuse.

19. Prior treatment with any anti-PD-1, or PD-L1 or PD-L2 agent; or prior treatment with azacitidine (any formulation) or any other hypomethylating agent.

20. Known or suspected hypersensitivity to azacitidine, pembrolizumab or the excipients of any of the study drugs. Known or suspected hypersensitivity to monoclonal antibodies.

21. Participation in the active part of other clinical trials of investigational agents in which last study treatment was administered within 4 weeks prior to registration in Part A or randomization in Part B.

22. Pregnant or lactating women.

### Procedures

This was an open-label, four-cohort, non-randomized study with administration of pembrolizumab (200 mg IV every 21 days) and oral 5-AZA at four dosing schedules: Cohort 1: 5-AZA 100 mg once daily on days 1–21; Cohort 2: 5-AZA 100 mg twice daily on days 1–21; Cohort 3: 5-AZA 300 mg once daily on days 1–14; Cohort 4: 5-AZA 300 mg once daily on days 1–21 (Supplementary Fig. 1). Eligible subjects were treated in one of four cohorts combined with pembrolizumab 200 mg intravenously on day 1. Subjects were assigned to a treatment cohort in the order in which they were enrolled in the study. Treatment was continued until unacceptable toxicity, progression of disease, consent withdrawal, or discontinuation by the investigator. Tumor assessment by irRECIST was completed 6 weeks after first administration of 5-AZA and then every 12 weeks thereafter.

### CA-125

Patients’ blood was collected for serum CA-125 at the discretion of the treating physician. Patients were considered evaluable for CA-125 assessment if the patient had a pre-treatment sample within 2 weeks prior to starting treatment, and an on-treatment or a post-treatment sample. Response for a patient was observed when there is at least a 50% reduction in CA-125 level from baseline.

### Target gene expression analyses

Tumor specimens were collected prior to treatment initiation and at 6 weeks on-therapy. Targeted transcriptomic analyses focused on direct digital counting of 770 unique genes using molecular barcodes

utilizing NanoString’s fluorescence-based direct digital detection chemistry (NanoString Technologies). Briefly, RNA was isolated from formalin-fixed paraffin-embedded (FFPE) tissue utilizing the Qiagen RNeasy FFPE kits (Qiagen). Isolated RNA was then hybridized to probes part of NanoString’s nCounter PanCancer IO 360 panel, which includes unique molecular barcodes for 770 immuno-oncology-related genes (NanoString Technologies). The probe set also included both positive and negative control probes; positive control probes were spiked into the probe panel and used to determine assay performance, whereas the negative control probes were designed as targets not expected to be present in the samples and used for background thresholding. A panel standard was included in each experiment for run-to-run comparisons. The nCounter MAX/FLEX system was used for sample processing: first, the nCounter Prep Station was used for post-hybridization purification of RNA and subsequent binding of the hybridized RNA onto nCounter cartridges. Cartridges were then placed on the digital analyzer to quantify gene expression. Raw gene expression counts were imported into the NanoString nSolver software for subsequent gene, pathway, and cell type analyses. Gene counts were normalized based on the geNorm algorithm, which selects for panel housekeeping genes that minimize the pairwise variation statistic (Supplementary Data 1–5). Target gene expression analyses we not performed in a dose-dependent manner because of the small number of cases per dosing cohort.

### RNA sequencing analyses

RNA sequencing was performed on RNA isolated from serial pre- and on-therapy tumor specimens collected before therapy initiation and 6 weeks after therapy initiation. Ribosomal RNA depletion was performed on total RNA using the Ribo-Zero Magnetic Gold Kit (Illumina). Subsequently, cDNA libraries were prepared and then sequenced on Illumina NovaSeq 6000 S4 (Illumina). Following sequencing, STAR was used for alignment of sequence reads to the reference genome^21^ and RSEM was used to quantify transcripts^22^. The quality of each individual sample was evaluated based on the total number of reads, the number of reads aligned to the reference genome, the number of reads aligned to the transcriptome, and the percentage of reads mapped to the genome that are also mapped to the transcriptome. Within each sequencing batch a universal human reference (UHR) sample was included as a control. Potential batch effects were evaluated through principal component analysis and a correlation of samples across the transcriptome, including the UHRs. Normalization of gene expression counts and differential expression was done utilizing DESeq2^23^. P-values from differential expression analysis were adjusted based on multiple testing corrections using the Benjamini-Hochberg procedure. Gene set enrichment analysis (GSEA) was performed utilizing the normalized gene expression counts, the fgsea (v.1.20.0) R package^24^, and a curated list of gene sets. Genes within each gene set were ranked by -log(P) * sign(fold-change). A list of the top 10 and bottom 10 gene sets for each comparison is shown in Supplementary Tables 1–3 and Supplementary Data 6–9. The TRUST4 algorithm was utilized to reconstruct TCR repertoires from RNAseq data^25^ (Supplementary Data 10). Within this analysis, we compared TCR clone dynamics for each individual patient with paired baseline and on-therapy tumor specimens as well as clone dynamics across CA-125 and clinical response labels. Individual TCR clones were considered significant if Fisher’s Exact p value < 0.01 when compared to the dynamics of all other clones for that patient.

### Statistics and reproducibility

Patient demographics and adverse events were summarized in descriptive tables. Efficacy data was analyzed based on the intention-to-treat population. The safety population consisted of all patients who received at least one dose of treatment. For nonparametric comparisons in the target gene expression and TCR data, the Wilcoxon rank-sum test was used. Statistical analyses were done with Python (version 3) or R version 3.6.1.

## Results

### Patient characteristics

**Fig. 1:**
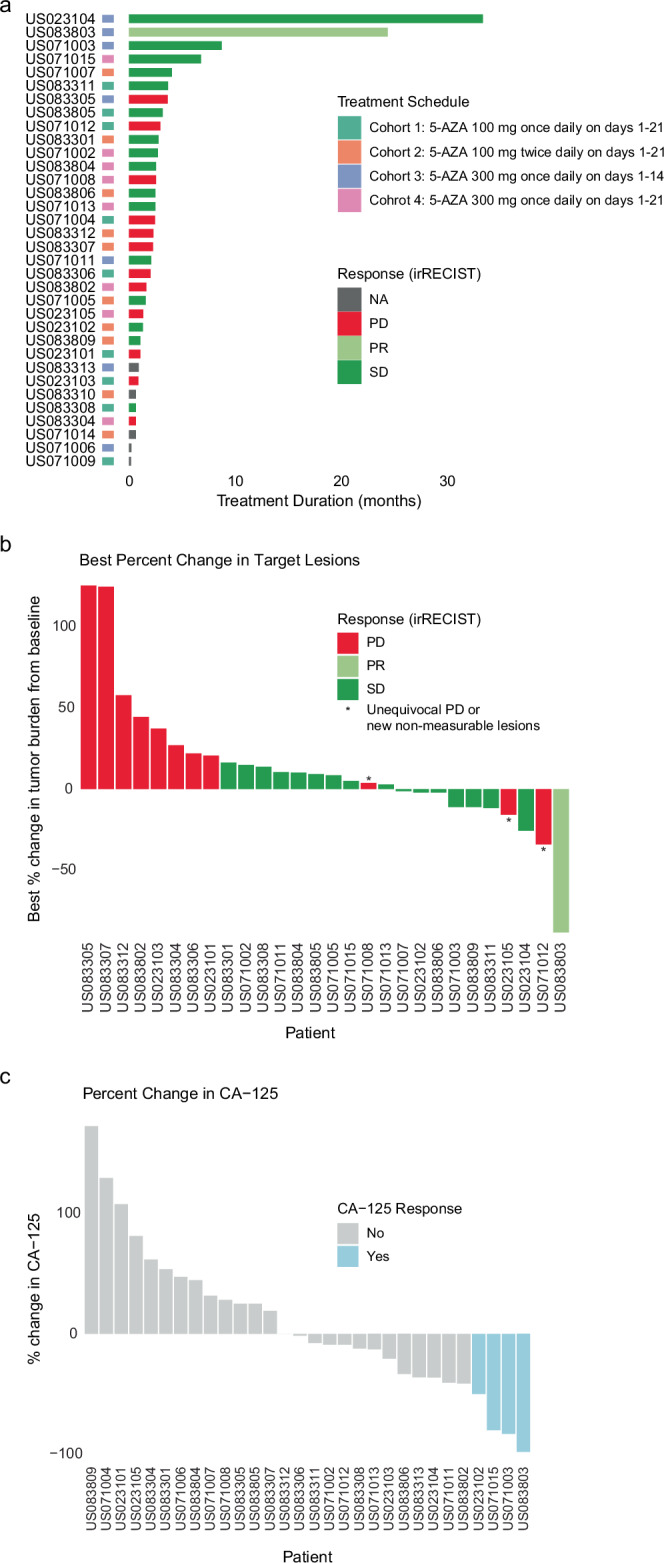
Radiographic and CA-125 responses in the study cohort. **a** Swimmer plot demonstrating treatment duration by patient in each cohort. (Cohort 1: 5-AZA 100 mg once daily on days 1–21; Cohort 2: 5-AZA 100 mg twice daily on days 1–21; Cohort 3: 5-AZA 300 mg once daily on days 1–14; Cohort 4: 5-AZA 300 mg once daily on days 1–21). **b** Waterfall plot showing tumor regression by irRECIST radiographic response assessment. An asterisk denotes patients who had less than a 20% increase in target lesions but had unequivocal PD due to new non-measurable target lesions. **c** Waterfall plot showing percent change in CA-125 from baseline to on-therapy for evaluable patients. Of the 27 evaluable patients, 3 attained a CA-125 response. 5-AZA azacitidine, PD progressive disease, PR partial response, SD stable disease.

A total of 34 patients were enrolled between January 2017 and August 2019 at four centers in the United States (Methods, Fig. 1a). Of the 34 patients in the intent to treat (ITT) population, 32 patients were in the safety population, and 20 patients were evaluable. A cohort remained open to accrual until five subjects treated on that schedule had completed two 5-AZA cycles and had the first post-baseline tumor burden assessment and both tumor biopsies performed and adequate paired tissue obtained. The baseline characteristics and demographics are summarized in Table 1. The median age was 63 years (range 32–78), and most patients (82.4%) had high-grade serous ovarian carcinoma. The majority of patients were heavily pre-treated, with most having more than 2 prior lines of therapy in the platinum-resistant/refractory setting.

### Trial design and endpoints

This phase II, open-label, multicenter study evaluated the safety and efficacy of combined oral 5-AZA in combination with pembrolizumab in patients with platinum-resistant/refractory epithelial ovarian cancer, fallopian tube cancer, or primary peritoneal cancer. The study enrolled four cohorts as follows: Cohort 1: 5-AZA 100 mg was administered once daily on days 1–21; Cohort 2: 5-AZA 100 mg was administered twice daily on days 1–21; Cohort 3: 5-AZA 300 mg once daily was administered on days 1–14; Cohort 4: 5-AZA 300 mg was administered once daily on days 1–21; all patients received pembrolizumab 200 mg intravenous on day 1 (Methods, Supplementary Fig. 1). Primary endpoints included safety, tolerability, overall response rate (ORR), and disease control rate (DCR) and secondary endpoints included CA-125 response (Methods). Transcriptomic analyses to understand therapy outcomes were exploratory endpoints.

### Safety and adverse events

All-grade adverse events (AEs) occurring in >20% of patients were vomiting (81.3%), nausea (75.0%), diarrhea (56.3%), anemia (40.6%), fatigue (37.5%), neutropenia (28.1%), aspartate aminotransferase (AST) increase (25%), and abdominal pain (21.9%); of these 16.9% were grade 3 or 4. The most common grade 3 or 4 adverse event was neutropenia, overall occurring in 8 patients (25%), with three patients (42.9%) in Cohort 3 and three patients (37.5%) in Cohort 4, compared to 0 patients in Cohort 1 and two patients (20%) in Cohort 2. Other grade 3 or 4 adverse events in the safety population included anemia (4 patients, 12.5%), fatigue (4 patients, 12.5%), elevated alanine aminotransferase (2 patients, 6.3%), vomiting (2 patients, 6.3%), diarrhea (1 patient, 3.1%), nausea (1 patient, 3.1%), AST elevation (1 patient, 3.1%), and headache (1 patient, 3.1%). Severe adverse events were reported in 14 (43.8%) of patients in the safety population. The most common severe adverse events were small bowel obstruction (SBO), neutropenia, and cerebrovascular accident (CVA). Neutropenia was related to 5-AZA, while SBO and CVA were deemed unrelated to 5-AZA or pembrolizumab. Adverse events are described in detail in Table 2.

Within the safety population, 13 patients (41%) required dose reductions in 5-AZA, most commonly due to neutropenia, nausea, and vomiting. Dose reductions in 5-AZA were more common in Cohort 3 (4 patients, 57.1%) and Cohort 4 (5 patients, 62.5%), compared to Cohort 1 (1 patient, 11.1%), and Cohort 2 (3 patients, 30%). Out of 34 patients in the intention-to-treat population, treatment was discontinued in 33 patients (97.1%), with 11 patients (32.4%) discontinuing due to an adverse event and 12 patients (35.3%) discontinuing due to disease progression. One patient in cohort 3 remained on study for 35 cycles, the maximum number of cycles allotted by the study. Specifically, by cohort, treatment was discontinued due to progressive disease according to irRECIST in five patients in Cohort 1 (55.6%), in two patients in Cohort 2 (20%), in one patient in Cohort 3 (14.3%), and in four patients in Cohort 4 (50%).

### Efficacy and response assessment

Patients completed a median of three cycles of therapy; efficacy is summarized in Table 1. One patient attained a partial response (2.9%), 16 patients had stable disease (47.1%), and 12 patients (35.3%) had progressive disease by irRECIST (Fig. 1a-b), resulting in an objective response rate (ORR) of 2.9%. The clinical benefit rate (CBR), defined as the fraction of patients who had a partial response or stable disease for at least six months, was 8.8%. DCR, defined as the fraction of patients with partial response and stable disease, was 50%. Notably, one patient with platinum-resistant clear cell ovarian cancer attained long-term stable disease and received 35 cycles of treatment (with an overall duration of treatment of 140 weeks). This patient completed the maximum number of cycles per study protocol and was subsequently switched to single-agent pembrolizumab; at the time of submission of this work, the patient remains in remission. Another patient with papillary serous platinum-refractory epithelial ovarian cancer attained a partial response and was on treatment for 27 cycles until disease progression.

### Assessment of CA-125 response

CA-125 response was evaluated as a secondary endpoint, and 27 patients were evaluable for CA-125 response assessments (Methods, Fig.1c). Three patients (8.8%) had a CA-125 response and attained a 50% reduction in pretreatment CA-125 values following therapy. Of these patients, one patient had a radiographic partial response, and 2 had radiographic stable disease.

### Transcriptomic analyses reveal an induction of inflammatory responses and reshaping of the tumor microenvironment with epigenetic therapy

**Fig. 2:**
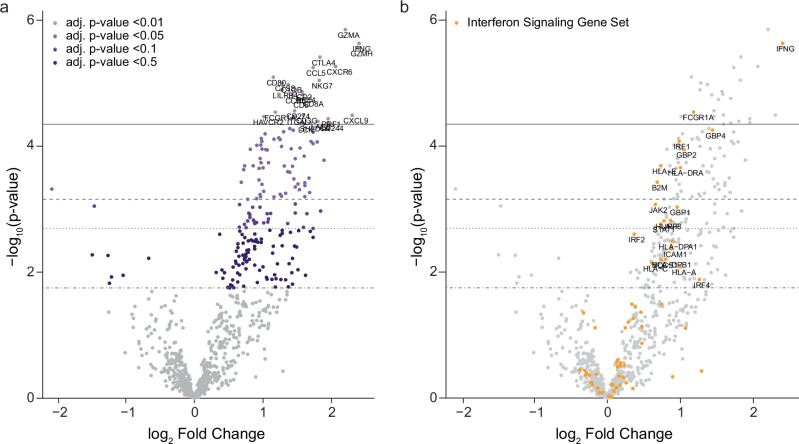
Differential enrichment of target immune response-related genes on-therapy. **a** Volcano plot depicting differential gene expression analyses revealing an upregulation of inflammatory and cytolytic genes as well as co-inhibitory checkpoints 6 weeks after treatment initiation (baseline *n* = 30 and on-therapy *n* = 18). **b** Volcano plot highlighting the interferon signaling gene set and upregulation of inflammatory and cytolytic genes. Differential expression analyses were performed utilizing NanoString’s nSolver Advanced Analysis Module with Benjamini-Yekutieli correction for multiple testing. Reporting FDR adjusted *p*-values.

**Fig. 3:**
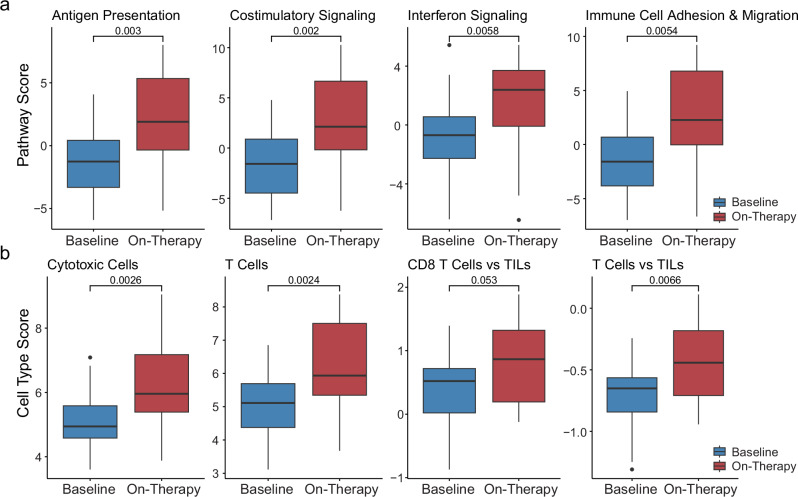
Pathway and cell type deconvolution based on serial target gene expression analyses. **a** Box plots of immune related pathways showing an upregulation of inflammatory pathways such as antigen presentation (baseline median pathway score −1.26; *n* = 30 vs on-therapy median pathway score 1.90; *n* = 18, two-sided Wilcoxon rank-sum test *p* = 0.003), costimulatory signaling (baseline median pathway score −1.57; *n* = 30 vs on-therapy median pathway score 2.12; *n* = 18, two-sided Wilcoxon rank-sum test *p* = 0.002), interferon signaling (baseline median pathway score -0.69; *n* = 30 vs on-therapy median pathway score 2.39; *n* = 18, two-sided Wilcoxon rank-sum test *p* = 0.0058), and immune cell adhesion and migration (baseline median pathway score -1.58; *n* = 30 vs on-therapy median pathway score 2.27; *n* = 18, two-sided Wilcoxon rank-sum test *p* = 0.0054) 6 weeks after therapy initiation. **b** Similarly, there is an upregulation of raw cytotoxic cell density (baseline median cell type score 4.94; *n* = 30 vs on-therapy median cell type score 5.96; *n* = 18, two-sided Wilcoxon rank-sum test *p* = 0.0026), raw T cell density (baseline median cell type score 5.11; *n* = 30 vs on-therapy median cell type score 5.94; *n* = 18, two-sided Wilcoxon rank-sum test *p* = 0.0024), relative CD8 + T cell-density (baseline median cell type score 0.52; *n* = 30 vs on-therapy median cell type score 0.87; *n* = 18, two-sided Wilcoxon rank-sum test *p* = 0.053), and relative T cell-density (baseline median cell type score −0.65 (*n* = 30) vs on-therapy median cell type score −0.44 (*n* = 18) two-sided Wilcoxon rank-sum test *p* = 0.0066) after therapy initiation.

We sought to evaluate the effect of epigenetic modulation in combination with immune checkpoint blockade through comprehensive transcriptomics analyses. As part of the study’s exploratory analyses, we performed direct digital detection of 770 unique genes on serially collected tumor biopsies to evaluate gene expression (Methods, Supplementary Data 1). Target gene expression analyses of 30 baseline and 18 on-therapy tumor specimens revealed differential enrichment of inflammatory and cytolytic genes (GZMA, IFNG, GZMH; False Discovery Rate (FDR)-adjusted *p* = 0.005) as well as the co-inhibitory molecule CTLA4 (FDR-adjusted *p* = 0.005) 6 weeks on-therapy compared to baseline (Fig. 2a-b, Supplementary Data 2). We then sought to understand the immune response on a broader level and provide additional context to the gene expression assessments through a pathway-level analysis. These analyses revealed an upregulation of antigen presentation, costimulatory signaling, interferon signaling, and immune cell adhesion and migration pathways on-therapy (Fig. 3a, Supplementary Data 3), indicating the induction of adaptive immune responses with epigenetic therapy.

We next determined whether there is a change in the immune cell composition in the TME of platinum-resistant ovarian tumors after treatment initiation. Using target gene expression data, we evaluated the density of each immune cell type based on the expression of their corresponding marker genes. We observed an increase in cytotoxic cells and T cells after initiation of combination therapy (Wilcoxon rank-sum test, *p*  =  0.003 and *p* = 0.002, respectively, Fig. 3b, Supplementary Data 3), indicating a heightened inflammatory response on-therapy. To confirm that the change in T cell density was not simply driven by an increase in the total number of cells, we determined the immune-related cellular composition based on the total amount of tumor-infiltrating lymphocytes (TILs). These analyses confirmed an increase in the number of total T cells and specifically CD8 + T cells relative to the total amount of TILs on-therapy (Wilcoxon rank-sum test, *p* = 0.007 and *p* = 0.05, respectively, Fig. 3b, Supplementary Data 3).

**Fig. 4:**
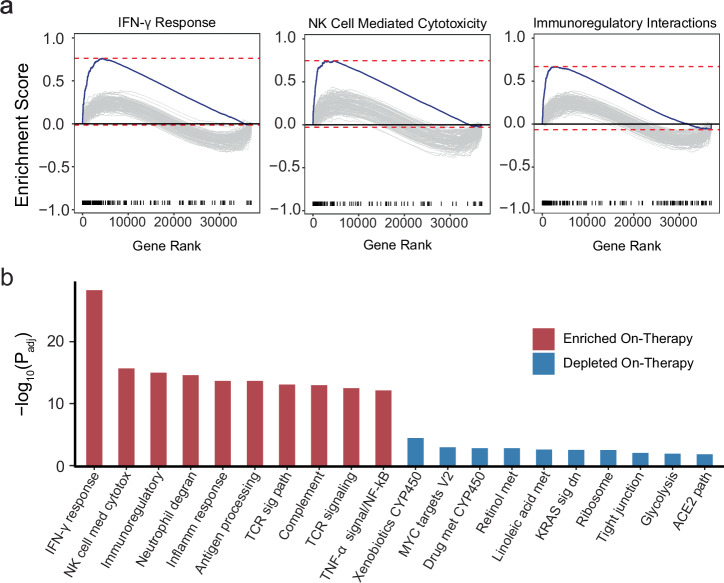
Gene set enrichment analyses of on-therapy tumors compared to baseline. GSEA leveraging RNA sequence data was concordant with the target gene analyses, showing an enrichment of key inflammatory gene sets 6 weeks after therapy initiation. **a** Enrichment plots of the interferon-γ, natural killer cell mediated cytotoxicity, and immunoregulatory interaction gene sets from baseline (*n* = 13) to 6 weeks after therapy initiation (*n* = 11). **b** Bar plot of the top 10 (red) most enriched genes sets and bottom 10 (blue) most depleted gene sets from baseline (*n* = 13) to 6 weeks after therapy initiation (*n* = 11). Two-sided gene set enrichment analysis and Benjamini–Hochberg correction for multiple testing used. Reporting FDR adjusted *p*-values.

We expanded target gene expression analyses, with an orthogonal whole-transcriptome approach in a subset of patients with available tumor tissue (13 baseline and 11 on-therapy tumor specimens). As part of these exploratory analyses, we performed bulk RNA sequencing (RNAseq) of serially collected tumor biopsies (Methods, Supplementary Table 1). Leveraging bulk RNAseq data, gene set enrichment analyses (GSEA) were performed to evaluate the differential expression of immune and inflammatory-related gene sets. GSEA was first performed with respect to timepoint, comparing pre-therapy to 6 weeks on-therapy. These analyses were concordant with target gene expression findings, showing an enrichment of immune and inflammatory gene sets at the on-therapy timepoint and an upregulation of gene sets linked with IFN-γ response, natural killer cell medicated cytotoxicity and immunoregulatory interactions (Fig. 4a-b, Supplementary Table 2). In addition to the enrichment of inflammatory related gene sets, we also found a depletion of the immunosuppressive MYC target gene set on-therapy (Fig. 4b). Our findings further support the induction of immune responses with combined epigenetic and immunotherapy that contribute to the reshaping of the tumor microenvironment.

To account for heterogeneity related to the different histological types, we excluded patients with endometrioid or clear cell ovarian carcinoma (*n* = 3), and performed target gene expression analyses in 27 baseline and 17 on-therapy specimens, focusing on patients with high-grade serous ovarian carcinoma (*n* = 27). In line with the analyses in the whole study population, we found an enrichment in inflammatory, cytolytic, and co-inhibitory genes on-therapy (Supplementary Fig. 2, and Supplementary Data 4). We found an increase in key immune-related pathways such as antigen presentation, costimulatory signaling, interferon signaling, and immune cell adhesion and migration pathways on-therapy, together with increased density of the T cell population, including cytotoxic and CD8 T cells (Supplementary Fig. 2, and Supplementary Data 5). Bulk RNA sequencing analyses in the same subset of patients (12 baseline and 10 on-therapy tumor specimens) were consistent with the analyses of the whole study cohort and showed a significant enrichment of inflammatory and adaptive immunity response  gene sets on-therapy (Supplementary Fig. 3, and Supplementary Table 3).

To further investigate TME reshaping on-therapy, we performed deconvolution of RNA sequencing data to infer T cell receptor (TCR) CDR3 assemblies by employing the TRUST4 algorithm (Methods). We focused these analyses on 11 cases with paired baseline and on-therapy tumor specimens. We explored individual TCR clone dynamics for each patient and evaluated significant TCR clonotypic expansions and regressions, indicative of reshaping of the TCR repertoire with combined epigenetic therapy and immunotherapy. We found that most intra-tumoral TCR repertoires were reshaped on-therapy, as evidenced by differential TCR abundance analyses (as shown for patient US083803 in Supplementary Fig. 4, Supplementary Data 10).

**Fig. 5:**
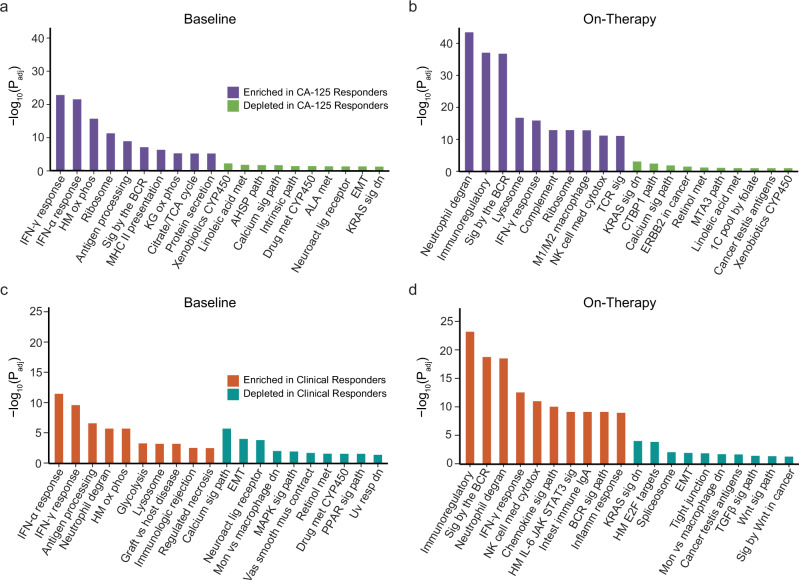
GSEA analyses based on CA-125 and clinical response. Patients with a CA-125 and/or clinical response showed an enrichment of adaptive and conserved immune response gene sets on-therapy. **a** Bar plot of the top 10 (purple) most enriched genes sets and bottom 10 (green) most depleted gene sets in CA-125 responders (*n* = 2) compared to non-CA-125 responders (*n* = 11) at baseline. **b** Bar plot of the top 10 (purple) most enriched genes sets and bottom 10 (green) most depleted gene sets in CA-125 responders (*n* = 2) compared to non-CA-125 responders (*n* = 9) 6 weeks after the initiation of therapy. **c** Bar plot of the top 10 (orange) most enriched genes sets and bottom 10 (blue) most depleted gene sets in clinical responders (*n* = 3) compared to non-clinical responders (*n* = 10) at baseline. **d** Bar plot of the top 10 (orange) most enriched genes sets and bottom 10 (blue) most depleted gene sets in clinical responders (*n* = 3) compared to non-clinical responders (*n* = 8) 6 weeks after the initiation of therapy. Two-sided gene set enrichment analysis and Benjamini–Hochberg correction for multiple testing used. Reporting FDR adjusted *p*-values. HM Hallmark, KG Kegg.

We then aimed to understand differential gene expression based on CA-125 and clinical response assessments (Methods, Supplementary Data 6, 7). Patients with a CA-125 response (*n* = 2) showed an enrichment of hallmark inflammatory gene sets (IFN-γ, IFN-α) at baseline compared to CA-125 non-responders (*n* = 11, Fig. 5a). Responding tumors (*n* = 2) showed an enrichment in adaptive and conserved immune response related gene sets after therapy initiation compared to non-responding tumors (*n* = 9, Fig. 5b). Similar to CA-125 responders, clinical responders (*n* = 3) also showed an enrichment of inflammatory related gene sets at baseline and adaptive immunity related gene sets on-therapy (Fig. 5c-d). These findings were replicated in restricting the analyses to the high-grade serous ovarian carcinoma subset (Supplementary Fig. 3, and Supplementary Data 8), suggesting that our observations do not appear to be confounded by histological subtype. Alternate patient grouping, assigning tumors with SD of any duration in the responding group, revealed similar findings with regard to enrichment of inflammatory gene sets in responding tumors on-therapy, though lessened in significance, supporting the original classification of clinical responders to exclude those with short-lived stable disease (Methods, Supplementary Data 9). In investigating the dynamics of differentially expanded or regressed TCR clones by CA-125 and clinical response, we observed a numerically higher -yet not statistically significant- number of significantly expanding and regressing clones for both CA-125 and clinical responders, providing further evidence of TME reshaping on-therapy (Supplementary Fig. 4). Collectively, the serial transcriptomic analyses showed an induction of inflammatory responses on-therapy after initiation of epigenetic therapy and immune checkpoint inhibition, especially in responding tumors.

## Discussion

Immunotherapy has demonstrated limited effectiveness for patients with platinum-resistant ovarian cancer, which is in-part related to an immunosuppressive tumor microenvironment (TME). Combination of epigenetic modulators and immune checkpoint inhibitors may reshape the TME of immunologically cold tumors and enhance anti-tumor immune responses by inducing viral mimicry. Here we report the safety and efficacy of combined epigenetic therapy and ICI together with comprehensive serial transcriptomic analyses aimed to capture the footprint of combination therapy in inducing an inflamed TME phenotype.

In this phase II study, the combination of pembrolizumab plus 5-AZA was moderately well tolerated, with the most common treatment-related adverse events being gastrointestinal (GI) side effects and neutropenia, which are well-known side effects of 5-AZA^26,27^. The higher rates of GI toxicity, specifically nausea and vomiting, noted in this study compared to 5-AZA in hematologic indications, may be attributed to the propensity of patients with advanced recurrent ovarian cancer to GI side effects in the context of disease extension to the GI tract and peritoneum, augmenting abdominal symptoms such as pain, nausea, and vomiting. Furthermore, oral 5-AZA administration is overall less tolerable compared to parenteral administration, with respect to GI toxicity. Cohorts 3 and 4, where higher dosages of 5-AZA were administered (300 mg daily), experienced higher treatment-related adverse events compared to Cohorts 1 and 2 and required dose reductions. Given the study design, adverse events could not be attributed separately to 5-AZA or pembrolizumab. No new safety signals were observed in any of the cohorts that would not be observed with monotherapy 5-AZA or pembrolizumab. Overall, combination therapy with pembrolizumab and 5-AZA showed overall safety and moderate tolerability, with better tolerability for lower 5-AZA dosing (Cohorts 1 and 2).

While combination of pembrolizumab with 5-AZA did not demonstrate an improvement in ORR compared to historic controls on pembrolizumab monotherapy -where overall response rates in the order of 8% have been reported^2^-, a number of patients had prolonged stable disease. The latter was reflected in a DCR rate of 50%, and two patients remained on treatment for longer than one year. The patients who remained on therapy the longest (27 cycles with a PR, and 35 cycles with SD) were both in Cohort 3. The limited clinical efficacy observed in our study may be partly due to the fact that the study cohort consisted of heavily pre-treated patients. Patients in this study had a high number of prior lines and over 40% had stage IV disease at diagnosis. Furthermore, over 20% of patients had primary platinum-refractory disease, which is typically an exclusion in immunotherapy clinical trials in ovarian cancer. These clinical characteristics are adverse prognostic markers and may have contributed to the low response rates seen in this study. The use of oral 5-AZA over subcutaneous 5-AZA may have also contributed, as pharmacodynamic studies have demonstrated that oral 5-AZA induces lower levels of DNA hypomethylation compared to subcutaneously administered 5-AZA^28^. The low overall response rate may also be driven by global hypomethylation of epithelial ovarian cancers, potentially rendering these tumors less sensitive to HMAs^29^. Nevertheless, given the limited therapy efficacy of standard of care treatments for patients with platinum-resistant/refractory epithelial ovarian cancer, prolonged disease stability suggests some clinical activity in this setting.

A similar study utilizing 5-AZA with durvalumab in advanced microsatellite stable colorectal cancer, platinum resistant ovarian cancer, and estrogen positive, HER2 negative breast cancer demonstrated similar ORR in all cohorts compared to our study, but had a lower disease control rate of 7.1%^27^. The DNA methylation profile in the patients of this basket trial showed minimal changes in tumor DNA methylation and a lack of ‘viral mimicry’ response^27^. In contrast, our study demonstrated a higher disease control rate of 50% together with induction of inflammatory responses in on-therapy tumors. Our findings are consistent with a previous phase II study of the DNA hypomethylating agent decitabine with carboplatin, which demonstrated that decitabine restored platinum-sensitivity in patients with PROC^18^. In that study, several pathways were found to be differentially methylated in patients who attained clinical benefit, including the JAK-STAT pathway and demethylation of tumor suppressor genes^18^.

Importantly, in our study, we assessed the biological footprint of combined anti-PD-1 therapy and epigenetic modulation on the tumor microenvironment by comprehensive serial transcriptomic analyses. 5-AZA has been shown to induce demethylation of endogenous retrovirus elements, resulting in activation of nucleic acid sensing pathway, ultimately triggering ‘viral mimicry’ and upregulation of type I interferon responses^9,10^. Consistent with this notion, we found an upregulation of inflammatory and cytolytic gene expression as well as interferon expression after initiation of therapy compared to baseline expression. These findings, while requiring further validation, suggest that the combination of epigenetic modulation and immunotherapy potentially contributes to reshaping of the TME towards a more inflamed phenotype.

The induction of an interferon response caused by epigenetic modulation has also been associated with a reversal of an immune exhausted phenotype through mechanisms such as the suppression of MYC signaling and increases in immune cell density and activation^11,12,16^. Within the bulk RNAseq data we observed a depletion of MYC related gene sets after the initiation of therapy and in the targeted gene expression data we observed an increase in the relative abundance of CD8 T cells on-therapy. Both observations are in line with the notion that type I interferon response initiated through epigenetic therapy causes a shift in immune cell populations in the TME and that the combination of epigenetic and immunotherapy may sensitize PROC to immunotherapy.

This study has several limitations, including the relatively small sample size for clinical and molecular analyses and the cohort heterogeneity with respect to different prior treatment regimens, and multiple histologies. While unlikely to confound the exploratory transcriptomic analyses, CA-125 response was defined as a CA-125 reduction of ≥ 50% from baseline assessment, which differs from the Gynecological Cancer Intergroup (GCIG)/Rustin definition, which requires confirmation after at least 28 days. Furthermore, this study did not provide direct evidence that 5-AZA had the intended effect of demethylation in tumor samples, which should be considered when interpreting our findings. Regarding the transcriptomic analyses, we note that the serial tumor biopsies were not always matched by anatomic location, which may introduce variability related to the differential composition of the tumor microenvironment in primary and metastatic lesions. Due to the number of cases per dosing cohort, we did not perform gene expression analyses in a dose-dependent manner.

As the trial design did not include a control arm of single-agent pembrolizumab, we could not definitively attribute the transcriptomic changes to epigenetic modulation alone. However, previous studies have shown that pembrolizumab alone has low anti-tumor activity in advanced ovarian cancer^2^ and have failed to detect an association between immune related gene expression programs and outcomes with immunotherapy, as seen with molecular analyses from the KEYNOTE-100 clinical trial^30^. These challenges may be circumvented by epigenetic modulation by DNA methyltransferase inhibitors, such as azacitidine, that have been shown to trigger an interferon response and may sensitize some tumors to immune checkpoint inhibition^9^. While our trial design did not allow for a separate evaluation of the biological effect of 5-AZA, epigenetic priming has been shown to induce immune response gene expression programs for patients with platinum-resistant ovarian cancer^14^. Future studies should focus on developing novel mechanisms to improve pharmacological efficacy of HMAs in combination with checkpoint inhibition and to use molecular signatures to personalize treatment for patients with platinum-resistance ovarian cancer. Additionally, future studies should investigate the dosing and scheduling of 5-AZA coupled with immunotherapy, together with a dose-dependent evaluation of transcriptomic changes in the tumor-microenvironment.

In conclusion, the combination of epigenetic modulation with 5-AZA and immune checkpoint inhibition was safe but moderately well tolerated, with a sizable portion of patients attaining disease control. Transcriptomic analyses revealed an upregulation of immune responses on-therapy, suggesting that combined epigenetic and immunotherapy shifts the tumor microenvironment towards a more inflamed phenotype, which is reflected in durable disease control for a fraction of patients with platinum-resistant ovarian cancers.

## Supplementary information

Supplementary Information

Description of Additional Supplementary Files

Supplementary Data 1

Supplementary Data 2

Supplementary Data 3

Supplementary Data 4

Supplementary Data 5

Supplementary Data 6

Supplementary Data 7

Supplementary Data 8

Supplementary Data 9

Supplementary Data 10

## Data Availability

The RNA-seq dataset is deposited to the European Genome-Phenome Archive (EGA; EGA accession EGAS50000001165 and EGA dataset EGAD50000001664). This dataset can be retrieved by request to the Data Access Committee (EGA DAC EGAC50000000697) through the EGA portal with completion of the data use agreement. Clinical trial data can be requested through the TRIO026 principal investigator at gkonecny@mednet.ucla.edu with an expected turnaround time of 4–8 weeks. Source data for the main figures of this manuscript are provided in the Supplementary Information and Supplementary Data files as follows: Source data for Fig. 2a-b is in Supplementary Data 2, source data for Fig. 3a-b is in Supplementary Data 3, source data for Fig. 4a-b is in Supplementary Table 2, source data for Fig. 5a-b is in Supplementary Data 6, source data for Fig. 5c-d is in Supplementary Data 7, source data for Supplementary Fig. 2a-b is in Supplementary Data 4, source data for Supplementary Fig. 2c-d is in Supplementary Data 5, source data for Supplementary Fig. 3a is in Supplementary Table 3, source data for Supplementary Fig. 3b-c is in Supplementary Data 8, source data for Supplementary Fig. 4a-c is in Supplementary Data 10.
