# Supplementary material for: Pembrolizumab and epigenetic modification with azacitidine reshapes the tumor microenvironment of platinum-resistant epithelial ovarian cancer: a phase 2 non-randomized clinical trial
Source: Commun Med (Lond). 2026 Feb 6;6:142. doi: 10.1038/s43856-026-01404-0 (PMC12988192; doi:10.1038/s43856-026-01404-0)
Supplement: Supplementary file 1 — Supplementary Information [file 43856_2026_1404_MOESM1_ESM.pdf]

Supplementary Information for

Pembrolizumab and epigenetic modification with azacitidine reshapes the tumor microenvironment of platinum-resistant epithelial ovarian cancer: a phase 2 non-randomized clinical trial

Blair V. Landon[1]*, Julia L. Boland[2]*, Andrea E. Wahner Hendrickson[3], Deborah K. Armstrong[1], Boris Winterhoff[4], Jaime Wehr[1], Akshaya V. Annapragada[1], Christopher Cherry[1], Archana Balan[1], Guneet Kaleka [5] Victor E. Velculescu[1], Stephen B. Baylin[1,6], Cynthia A. Zahnow[1], Dennis J. Slamon[7], Gottfried E. Konecny[7], Valsamo Anagnostou[1#] and John A. Glaspy[7#]

1. Department of Oncology, Sidney Kimmel Comprehensive Cancer Center, Johns Hopkins University School of Medicine, Baltimore, MD, USA
2. Department of Medicine, Division of Internal Medicine, David Geffen School of Medicine, University of California Los Angeles, Los Angeles, CA, USA
3. Department of Medical Oncology, Mayo Clinic, Rochester, MN, USA
4. Department of Obstetrics and Gynecology, University of Minnesota, Minneapolis, MN, USA
5. Department of Medicine, UCLA-Olive View Medical Center, Department of Medicine, Sylmar, CA, USA
6. Department of Epigenetics, Van Andel Institute, Grand Rapids, MI, USA
7. Department of Medicine, Division of Hematology/Oncology, David Geffen School of Medicine, University of California Los Angeles, Los Angeles, CA, USA

*Authors contributed equally
[#]Co-senior authors

Correspondence should be addressed to:

John Glaspy MD
University of California Los Angeles
100 Medical Plaza Driveway
Suite 550
Los Angeles, CA 90095
Tel: +1 310-794-4955
Email: jglaspy@mednet.ucla.edu

and
Valsamo Anagnostou, MD, PhD
Sidney Kimmel Comprehensive Cancer Center
Cancer Research Building 2, Rm 546
1550 Orleans Street, Baltimore, MD, 21287
Tel: +1 410-614-8948
Email: vanagno1@jhmi.edu

<u>Supplementary Figures</u>

Supplementary Figure 1: CONSORT diagram and cohort treatment and biospecimen collection schema. Page 3

Supplementary Figure 2: Target gene differential enrichment, pathway and cell type deconvolution including only specimens derived from patients with high-grade serous carcinoma. Page 5

Supplementary Figure 3: Gene set enrichment analyses across therapy and response labels focusing on specimens derived from patients with high-grade serous carcinoma. Page 7

Supplementary Figure 4: T cell receptor (TCR) repertoire reshaping. Page 9

<u>Supplementary Tables</u>

Supplementary Table 1: Summary of bulk RNA sequencing metrics of tumors analyzed. Page 11

Supplementary Table 2: Bulk RNAseq gene set enrichment analysis for on-therapy vs baseline tumors. Page 12

Supplementary Table 3: Bulk RNAseq gene set enrichment analysis in on-therapy vs baseline tumors, focusing on high-grade serous carcinomas. Page 13

Supplementary Figures

a

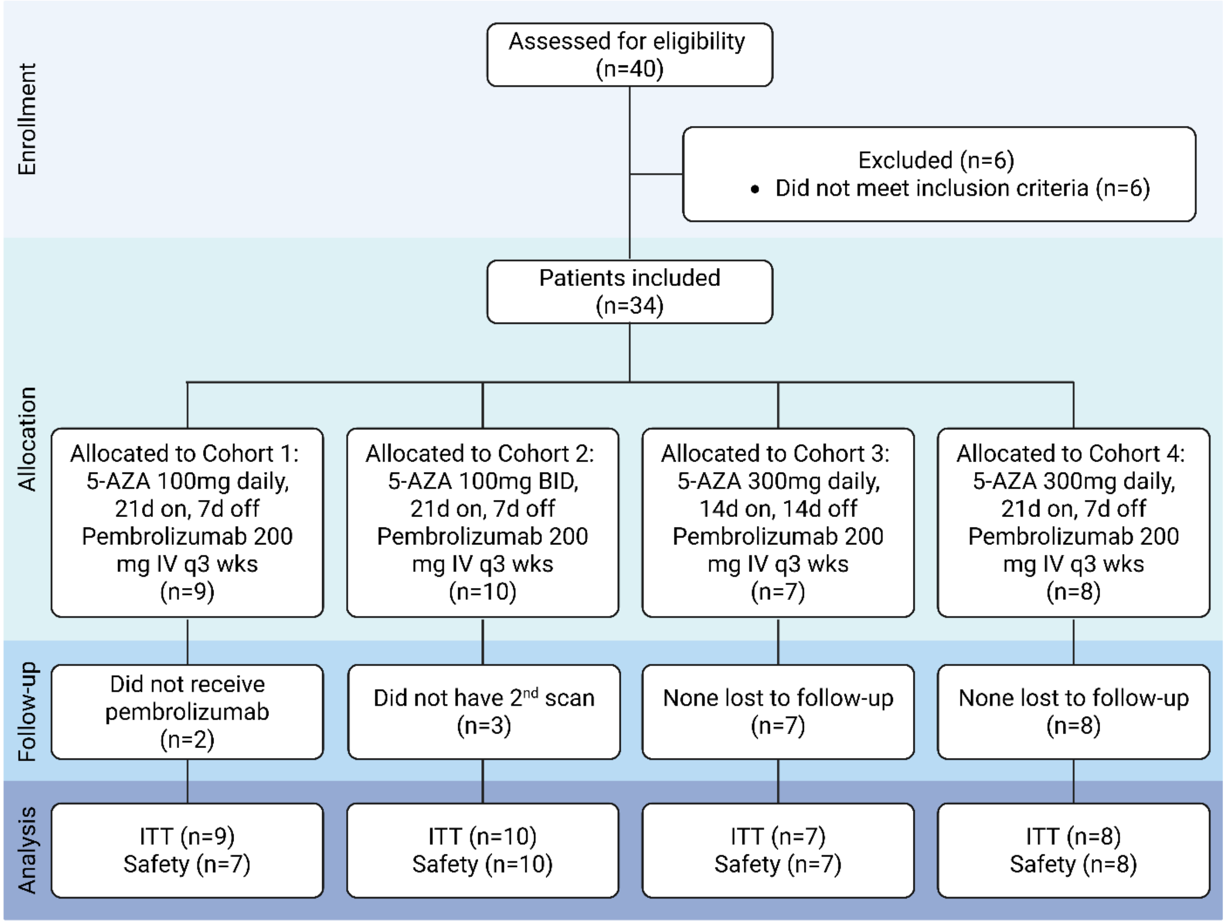

b

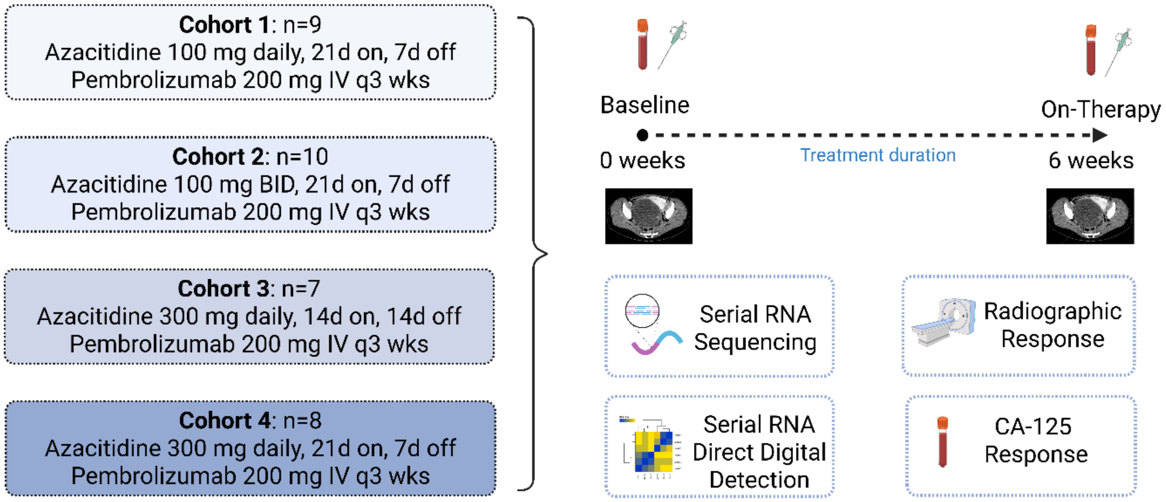

Supplementary Figure 1. CONSORT diagram and cohort treatment and biospecimen collection schema. **(a)** CONSORT diagram depicting patient disposition. Patients with epithelial ovarian cancer (EOC), fallopian tube carcinoma or primary peritoneal carcinoma were enrolled in an open-label, non-randomized, four-cohort study to receive pembrolizumab (200 mg of IV every 21 days) and oral azacitidine, 5-AZA. The four cohorts varied based on dosing of 5-AZA: Cohort 1: 5-AZA 100 mg once daily on days 1-21; Cohort 2: 5-AZA 100 mg twice daily on days 1-21; Cohort 3: 5-AZA 300 mg once daily on days 1-14; Cohort 4: 5-AZA 300 mg once daily on days 1-21. **(b)** Tumor specimens were collected before the start of therapy and at 6 weeks after initiation of therapy. Bulk RNA sequencing and target gene expression analysis was performed on serial tumor specimens. Patients were classified as responders based on radiographic imaging and CA-125 levels. BID, twice daily; ITT, intention-to-treat; 5-AZA, azacitidine. This figure was generated in BioRender under a paid individual license; Landon, B. (2025) https://BioRender.com/6y3e4cr.

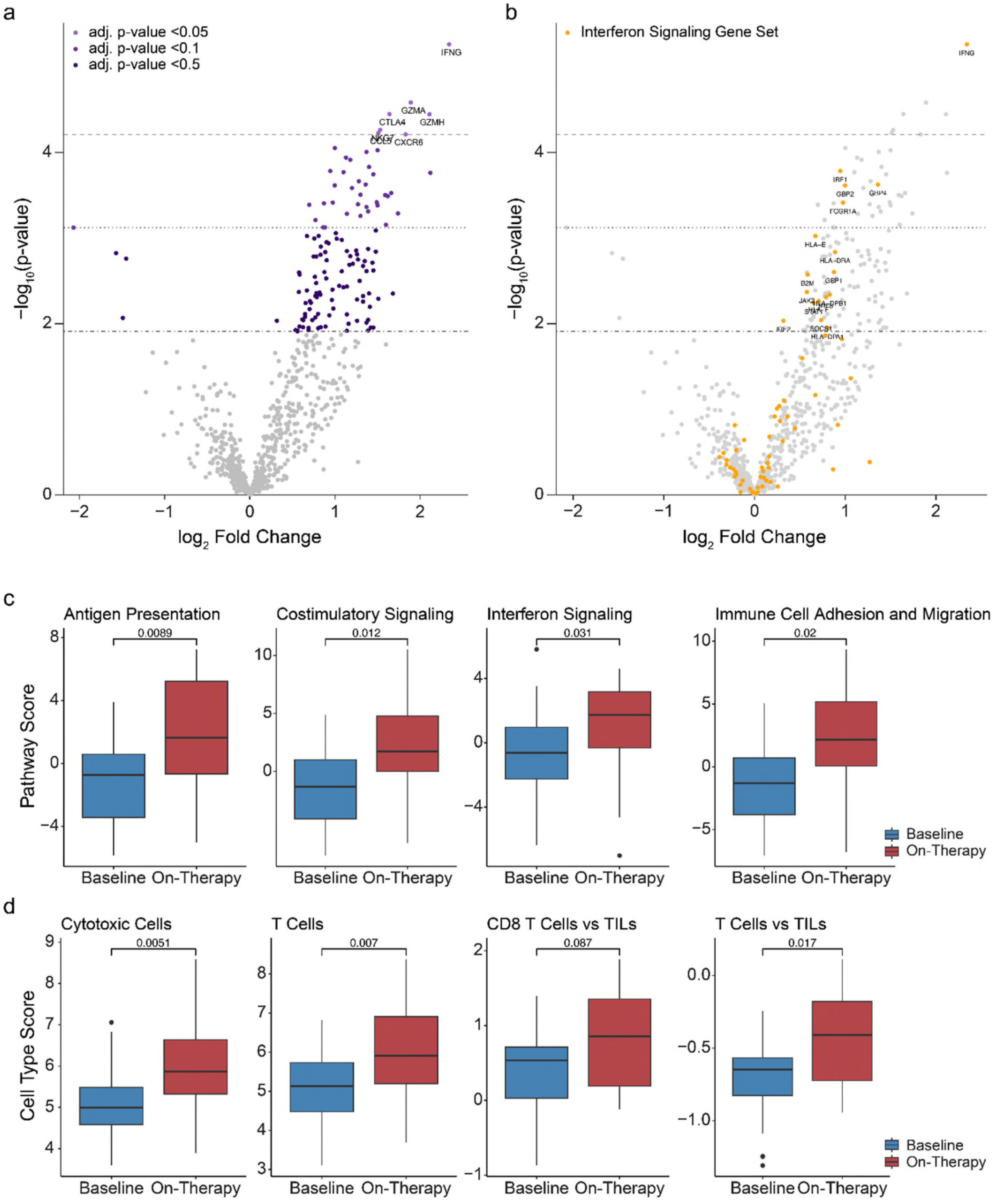

Supplementary Figure 2. Target gene differential enrichment, pathway and cell type deconvolution including only specimens derived from patients with high-grade serous carcinoma. (a) Volcano plot representing differential enrichment of immune and inflammatory genes on-therapy (baseline n=27 and on-therapy n=17). **(b)** Volcano plots focusing only on genes in the interferon signaling gene set (yellow).

Differential expression analyses were performed utilizing NanoString's nSolver Advanced Analysis Module with Benjamini-Yekutieli correction for multiple testing. Reporting FDR adjusted p-values. **(c)** Box plots depicting comparisons of immune and inflammatory pathways at baseline and six weeks on-therapy: antigen presentation (baseline median pathway score -0.74; n=27 vs on-therapy median pathway score 1.62; n=17, two-sided Wilcoxon rank-sum test p=0.0089), costimulatory signaling (baseline median pathway score -1.33; n=27 vs on-therapy median pathway score 1.73; n=17, two-sided Wilcoxon rank-sum test p=0.012), interferon signaling (baseline median pathway score -0.63; n=27 vs on-therapy median pathway score 1.73; n=17, two-sided Wilcoxon rank-sum test p=0.031), and immune cell adhesion and migration (baseline median pathway score -1.27; n=27 vs on-therapy median pathway score 2.17; n=17, two-sided Wilcoxon rank-sum test p=0.02). **(d)** Box plots representing baseline and on-therapy comparisons for raw and relative cell types scores of relevant immune cell populations: raw cytotoxic cell density (baseline median cell type score 4.99; n=27 vs on-therapy median cell type score 5.87; n=17, two-sided Wilcoxon rank-sum test p=0.0051), raw T cell density (baseline median cell type score 5.13; n=27 vs on-therapy median cell type score 5.91; n=17, two-sided Wilcoxon rank-sum test p=0.007), relative $CD8^+$ T cell density (baseline median cell type score 0.54; n=27 vs on-therapy median cell type score 0.86; n=17, two-sided Wilcoxon rank-sum test p=0.087), and relative T cell density (baseline median cell type score -0.65; n=27 vs on-therapy median cell type score -0.41; n=17, two-sided Wilcoxon rank-sum test p=0.017).

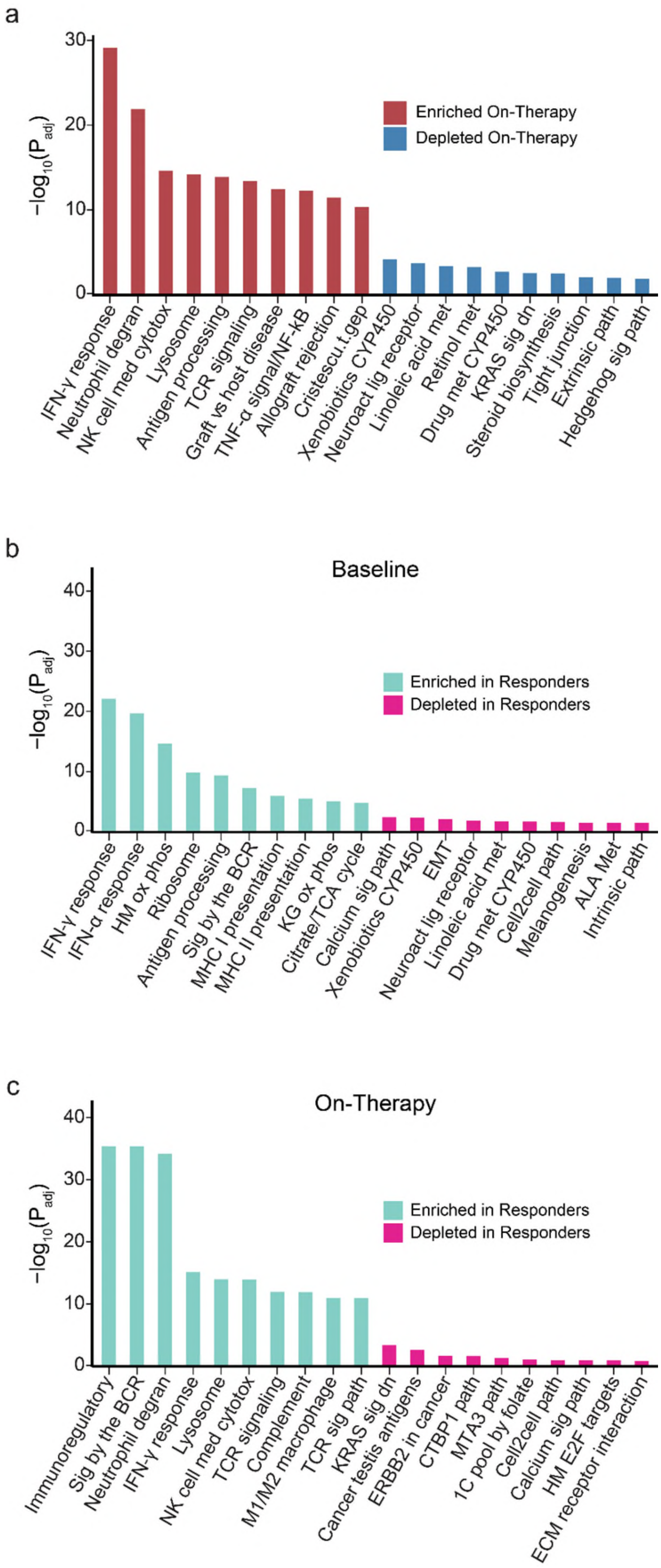
a
-log10(Padj)
Enriched On-Therapy
Depleted On-Therapy
IFN-γ response
Neutrophil degran
NK cell med cytotox
Lysosome
Antigen processing
TCR signaling
Graft vs host disease
TNF-α signal/NF-kB
Allograft rejection
Cristescu.t.gep
Xenobiotics CYP450
Neuroact lig receptor
Linoleic acid met
Retinol met
Drug met CYP450
KRAS sig dn
Steroid biosynthesis
Tight junction
Extrinsic path
Hedgehog sig path
b
Baseline
-log10(Padj)
Enriched in Responders
Depleted in Responders
IFN-γ response
IFN-α response
HM ox phos
Ribosome
Antigen processing
Sig by the BCR
MHC I presentation
MHC II presentation
KG ox phos
Citrate/TCA cycle
Calcium sig path
Xenobiotics CYP450
EMT
Neuroact lig receptor
Linoleic acid met
Drug met CYP450
Cell2cell path
Melanogenesis
ALA Met
Intrinsic path
c
On-Therapy
-log10(Padj)
Enriched in Responders
Depleted in Responders
Immunoregulatory
Sig by the BCR
Neutrophil degran
IFN-γ response
Lysosome
NK cell med cytotox
TCR signaling
Complement
M1/M2 macrophage
TCR sig path
KRAS sig dn
Cancer testis antigens
ERBB2 in cancer
CTBP1 path
MTA3 path
1C pool by folate
Cell2cell path
Calcium sig path
HM E2F targets
ECM receptor interaction

Supplementary Figure 3. Gene set enrichment analyses across therapy and response labels focusing on specimens derived from patients with high-grade serous carcinoma. (a) Bar plot of GSEA comparing baseline and on-therapy. The top 10 most enriched (red) and bottom 10 most depleted (blue) gene sets on-therapy (n=10) compared to baseline (n=12). **(b)** Bar plot representing GSEA of top 10 most enriched (aqua) and bottom 10 most depleted (pink) gene sets comparing responders (n=2) to non-responders (n=10) at baseline. **(c)** Bar plot representing GSEA of top 10 most enriched (aqua) and bottom 10 most depleted (pink) gene sets comparing clinical responders (n=2) to non-clinical responders (n=8) 6 weeks after initiation of therapy. Two-sided gene set enrichment analysis and Benjamini–Hochberg correction for multiple testing used. Reporting FDR adjusted p-values.

*The removal of 1 patient with clear cell carcinoma who did not attain a CA-125 response, but attained a clinical response resulted in the CA-125 and clinical response labels being the same. Thus, the term responders is labeled in panels b-c.

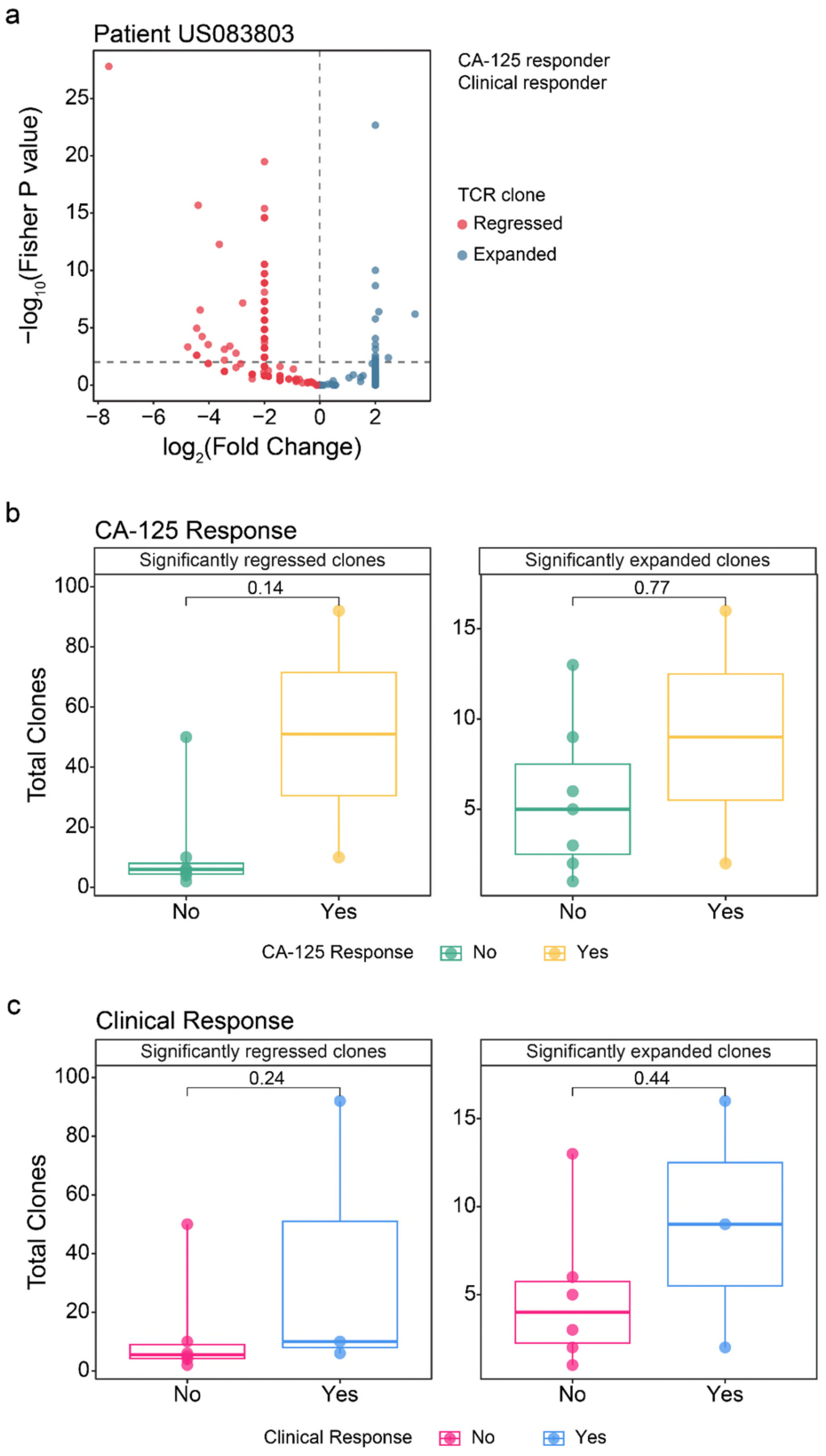
a
Patient US083803
CA-125 responder
Clinical responder
TCR clone
Regressed
Expanded
−log10(Fisher P value)
log2(Fold Change)
b
CA-125 Response
Significantly regressed clones
Significantly expanded clones
0.14
0.77
Total Clones
No
Yes
CA-125 Response
c
Clinical Response
Significantly regressed clones
Significantly expanded clones
0.24
0.44
Total Clones
No
Yes
Clinical Response

Supplementary Figure 4. T cell receptor (TCR) repertoire reshaping. (a) TCR clone dynamics for patient US083803, who attained both a CA-125 and clinical response. This patient had a number of expanded (blue) and regressed (red) clones when comparing from baseline to 6 weeks after the initiation of therapy, suggesting reshaping of the tumor microenvironment. **(b)** Box plots of total significant TCR clone counts per patient stratified by CA-125 response and direction of TCR clones dynamics (expanded or regressed). Significantly regressed clones were numerically higher in CA-125 responders vs non-CA-125 responders (non-CA-125 responders median total clone count 6; n=7 vs CA-125 responders median total clone count 51; n=2, two-sided Wilcoxon rank-sum test p=0.14). There was no difference in significantly expanded clones in CA-125 responders vs non-CA-125 responders (non-CA-125 responders median total clone count 5; n=7 vs CA-125 responders median total clone count 9; n=2, two-sided Wilcoxon rank-sum test p=0.77. **(c)** Box plots of total TCR clones per patient stratified by clinical response and direction of TCR clone dynamics (expanded or regressed). Significantly regressed clones were numerically higher in clinical responders vs non-clinical responders (non-clinical responders median total clone count 5.5; n=6 vs clinical responders median total clone count 10; n=3, two-sided Wilcoxon rank-sum test p=0.24). There was no difference in significantly expanded clones in clinical responders vs non-clinical responders (non-clinical responders median total clone count 4; n=6 vs clinical responders median total clone count 9; n=3, two-sided Wilcoxon rank-sum test p=0.44). Both response labels show a numerical difference in significantly regressed clone counts between responders and non-responders. TCR clones are considered significant based on a Fisher's p value <0.01 for their increase or decrease compared to all other clones in the same

Supplementary Tables

Supplementary Table 1: Summary of bulk RNA sequencing metrics of tumors analyzed.

Specimen ID	Timepoint	Sequenced Reads	Reads After Trimming	Reads Mapped To Genome	Reads Mapped To Transcriptome
US023105_FFTBL	Baseline	222668072	158487968	138893532	55149298
US023105_FFTC2	On-Therapy	266424998	196035182	177182879	70887110
US071002_FFTBL	Baseline	200130508	143293524	120202851	49740758
US071002_FFTC2	On-Therapy	210909368	152012048	126293667	57958898
US071013_FFTBL	Baseline	201361748	150320264	133711680	58588818
US071013_FFTC2	On-Therapy	202633624	148268248	131152412	49904636
US071015_FFTBL	Baseline	227552190	166848018	142575310	64500286
US071015_FFTC2	On-Therapy	200527234	142412182	122696799	66924250
US083301_FFTBL	Baseline	258141824	188343604	168995741	64598962
US083301_FFTC2	On-Therapy	247626212	186865054	166793312	85791546
US083305_FFTBL	Baseline	216243252	159988154	142535708	70082056
US083305_FFTC2	On-Therapy	204605714	147216244	120557710	53937880
US083307_FFTBL	Baseline	200219778	145196078	127158466	55757426
US083307_FFTC2	On-Therapy	201749330	143166652	126148974	50878798
US083312_FFTBL	Baseline	398268878	308756800	266744360	136920874
US083312_FFTC2	On-Therapy	488305506	386689230	351404477	196718940
US083802_FFTBL	Baseline	200782160	147468794	129047034	70174414
US083802_FFTC2	On-Therapy	201342676	143259652	124902113	68299674
US083803_FFTBL	Baseline	214751742	161167320	140038700	91795344
US083803_FFTC2	On-Therapy	220158742	156531298	127835676	75012844
US023104_FFTBL	Baseline	206824482	133752828	116459510	37663436
US023104_FFTC2	On-Therapy	203985108	80641748	64351769	23339146
US083304_FFTBL	Baseline	202337992	128596806	107766052	44567524
US083308_FFTBL	Baseline	203506294	135484826	118709140	60055782

Supplementary Table 2: Bulk RNAseq gene set enrichment analysis for on-therapy vs baseline tumors.

Gene Set Shortened Name	Gene Set Original Name	P value (adjusted)	Normalized Enrichment Score	Gene Set Size
IFN-γ response	Hm Interferon Gamma Resp	4.86881E-29	2.678488853	198
NK cell med cytotox	Kg Natural Killer Cell Mediated Cytotoxicity	2.09882E-16	2.482467212	123
Immunoregulatory	Rt Immunoregulatory Interactions Between A Lymphoid And A Non Lymphoid Cell	1.03288E-15	2.337841607	185
Neutrophil degran	Rt Neutrophil Degranulation	2.62281E-15	2.082728581	461
Inflamm response	Hm Inflammatory Resp	2.16525E-14	2.285945631	197
Antigen processing	Kg Antigen Processing And Presentation	2.16525E-14	2.518861888	71
TCR sig path	Kg T Cell Receptor Sig Path	8.57058E-14	2.419287355	107
Complement	Hm Complement	1.05946E-13	2.279485704	199
TCR signaling	Rt Tcr Sig	3.17961E-13	2.390495003	118
TNF-α signal/NF-κB	Hm Tnfa Sig Via Nfkb	7.59151E-13	2.240649455	198
Xenobiotics CYP450	Kg Met Of Xenobiotics By Cytochrome P450	3.61929E-05	-2.082311146	69
MYC targets V2	Hm Myc Targets V2	0.001134482	-1.886962872	58
Drug met CYP450	Kg Drug Met Cytochrome P450	0.001589975	-1.823570636	71
Retinol met	Kg Retinol Met	0.001597979	-1.88045225	62
Linoleic acid met	Kg Linoleic Acid Met	0.002598942	-1.940317437	29
KRAS sig dn	Hm Kras Sig Dn	0.003024503	-1.550895662	188
Ribosome	Kg Ribosome	0.003213194	-1.708805808	85
Tight junction	Kg Tight Junction	0.009189891	-1.568499608	127
Glycolysis	Hm Glycolysis	0.012175909	-1.430280269	197
ACE2 path	Bc Ace2 Path	0.01490255	-1.79620122	13

Supplementary Table 3: Bulk RNAseq gene set enrichment analysis in on-therapy vs baseline tumors, focusing on high-grade serous carcinomas.

Gene Set Shortened Name	Geneset Original Name	P value (adjusted)	Normalized Enrichment Score	Gene set size
IFN-γ response	Hm Interferon Gamma Resp	6.8969E-30	2.807717343	198
Neutrophil degran	Rt Neutrophil Degranulation	1.22213E-22	2.320545624	461
NK cell med cytotox	Kg Natural Killer Cell Mediated Cytotoxicity	2.6152E-15	2.539246861	123
Lysosome	Kg Lysosome	6.62573E-15	2.502190421	121
Antigen processing	Kg Antigen Processing And Presentation	1.32577E-14	2.572432944	71
TCR signaling	Rt Tcr Sig	3.98292E-14	2.457147893	118
Graft vs host disease	Kg Graft Versus Host Disease	3.45912E-13	2.522567273	38
TNF-α signal/NF-κB	Hm Tnfa Sig Via Nfkb	5.78077E-13	2.293768225	198
Allograft rejection	Kg Allograft Rejection	3.69529E-12	2.534483645	35
Cristescu.t.gep	Cristescu.t.gep	4.80241E-11	2.316661962	18
Xenobiotics CYP450	Kg Met Of Xenobiotics By Cytochrome P450	6.91E-05	-2.05E+00	69
Neuroactive lig receptor	Kg Neuroactive Ligand Receptor Interaction	0.000199943	-1.696765347	256
Linoleic acid met	Kg Linoleic Acid Met	0.000446201	-2.014997782	29
Retinol met	Kg Retinol Met	0.000609806	-1.924216205	62
Drug met CYP450	Kg Drug Met Cytochrome P450	0.002092018	-1.797143792	71
KRAS sig dn	Hm Kras Sig Dn	0.00307089	-1.596436552	188
Steroid biosynthesis	Kg Steroid Hormone Biosynthesis	0.003214297	-1.804977453	55
Tight junction	Kg Tight Junction	0.009184552	-1.577222579	127
Extrinsic path	Bc Extrinsic Path	0.010591629	-1.78834703	13
Hedgehog sig path	Kg Hedgehog Sig Path	0.014911239	-1.688904234	56
