# Supplementary material for: Pembrolizumab and epigenetic modification with azacitidine reshapes the tumor microenvironment of platinum-resistant epithelial ovarian cancer: a phase 2 non-randomized clinical trial
Source: Commun Med (Lond). 2026 Feb 6;6:142. doi: 10.1038/s43856-026-01404-0 (PMC12988192; doi:10.1038/s43856-026-01404-0)
Supplement: Supplementary file 2 — Description of Additional Supplementary Files [file 43856_2026_1404_MOESM2_ESM.pdf]

Description of Additional Supplementary Files

File name: Supplementary Data 1

Description: Short summary of all tumor specimens analysed in the target gene expression analyses.

File name: Supplementary Data 2

Description: Contains all data from target gene expression analysis comparing baseline expression of each gene to on-therapy expression. All baseline samples across patients are grouped together and all on-therapy samples are grouped together. Data is organized by gene.

File name: Supplementary Data 3

Description: Contains all pathway and cell type scores derived from comparing baseline to on-therapy expression. Data is organized by sample.

File name: Supplementary Data 4

Description: Contains all data from target gene expression analysis comparing baseline expression of each gene to on-therapy expression only in specimens derived from patients with high-grade serous carcinoma. All baseline samples across patients are grouped together and all on-therapy samples are grouped together. Data is organized by gene.

File name: Supplementary Data 5

Description: Contains all pathway and cell type scores derived from comparing baseline to on-therapy expression only in specimens derived from patients with high-grade serous carcinoma. Data is organized by sample.

File name: Supplementary Data 6

Description: Contains a list of the top 10 and bottom 10 gene sets at baseline and on-therapy when comparing CA-125 responders to CA-125 non-responders. Data is organized by gene set timepoint and direction.

File name: Supplementary Data 7

Description: Contains a list of the top 10 and bottom 10 gene sets at baseline and on-therapy when comparing clinical responders to clinical non-responders. Data is organized by gene set timepoint and direction.

File name: Supplementary Data 8

Description: Contains a list of the top 10 and bottom 10 gene sets at baseline and on-therapy when comparing responders to non-responders, focusing only on specimens derived from patients with high-grade serous carcinoma. Data is organized by gene set timepoint and direction.

File name: Supplementary Data 9

Description: Contains a list of the top 10 and bottom 10 gene sets at baseline and on-therapy when comparing tumor specimens derived from patients attaining either partial response, durable stable disease, or stable disease to tumor specimens derived from patients with progressive disease. Data is organized by gene set timepoint and direction.

File name: Supplementary Data 10

Description: Contains all inferred T cell receptor (TCR) CDR3 assemblies from the deconvolution of RNAseq data. Table is organized by patient and then list clones for each patient.
